# Cataract Prevalence in Patients with Cutaneous Melanoma in Lithuanian Population

**DOI:** 10.3390/jcm13226717

**Published:** 2024-11-08

**Authors:** Lukas Šemeklis, Laura Kapitanovaitė, Grinvydas Butrimas, Kamilija Briedė, Laura Račkauskaitė, Reda Žemaitienė, Skaidra Valiukevičienė

**Affiliations:** 1 Department of Ophthalmology, Medical Academy, Lithuanian University of Health Sciences, 44037 Kaunas, Lithuania; 2 Department of Ophthalmology, Hospital of Lithuanian University of Health Sciences Kauno Klinikos, 50161 Kaunas, Lithuania; 3 Department of Skin and Venereal Diseases, Medical Academy, Lithuanian University of Health Sciences, 44037 Kaunas, Lithuania; 4 Department of Skin and Venereal Diseases, Hospital of Lithuanian University of Health Sciences Kauno Klinikos, 50161 Kaunas, Lithuania

**Keywords:** sun exposure, ultraviolet radiation, cutaneous melanoma, cataract

## Abstract

**Background/Objectives:** Sun exposure and ultraviolet (UV) radiation significantly affect human health, especially concerning skin and eye conditions. Sun exposure is a risk factor for both cutaneous melanoma (CM) and cataract. To investigate the association between CM, cataract and the number of common melanocytic nevi (CMNs) in the Lithuanian population. **Methods:** A case–control study with 180 primary diagnosed CM subjects and 182 healthy controls was conducted. Participants underwent ophthalmic and dermatological examination, where a counting of the common melanocytic nevi (CMNs) on the face, outer surfaces of the upper arms, lower arms and hands was performed. A detailed ophthalmic slit lamp examination was conducted; additionally, lens status and cataract formation were evaluated according to the Lens Opacities Classification System III (LOCS III). **Results**: Subjects with an LOCS III grade of nuclear opalescence (NO) ≥3 had a 1.82 times higher risk of CM, and patients with a nuclear color (NC) grade ≥3 had a 2.02 times higher risk of CM. LOCS III evaluations of cortical (C) and of posterior subcapsular (P) cataract showed a 5.24 and 6.34 times increased risk of CM, respectively. The CMN number on the face increased CM risk by 1.25 times; on the outer surface of the upper and lower arms, correspondingly 1.05 and 1.04 times; and on outer surface of hands—1.29 times. **Conclusions**: All types of cataracts were found more often in patients with CM than in healthy subjects. The number of CMNs on the face and outer surfaces of arms and hands could be an indicator of higher risk for CM.

## 1. Introduction

Sun exposure significantly affects human health especially concerning skin and eye conditions. Ultraviolet radiation (UV) is part of the sun’s spectrum, and it is divided into three categories based on wavelength: UVA (320–400 nm), UVB (290–320 nm) and UVC (100–290 nm). Among these types, UVA and UVB are most relevant to health as UVC is mostly absorbed by Earth’s atmosphere. UVA can penetrate deeper into the skin while UVB is primarily responsible for causing sunburn and direct DNA damage [1]. Both forms of UV radiation significantly contribute to the development of cutaneous melanoma (CM) and cataract through processes involving oxidative stress and cellular harm [2,3].

CM is the most aggressive skin cancer. Sunbeds and indoor tanning, phenotypic characteristics such as fair skin and hair, light eye color and freckles and a high number of common and atypical melanocytic nevi are all risk factors for CM [4,5].

Additionally, in our previous study, we found that an iris pattern with a blue or gray periphery and a blue collaret with iris freckles, as well as the presence of pigmented iris lesions, are associated with a higher risk of CM [6].

Cataract is the opacification of the lens and is a leading cause of blindness worldwide. Cataract accounts for around a third of all cases of blindness, globally affecting 100 million individuals [7]. The human lens absorbs the majority of UVB exposure, and it leads to the formation of cataract [1,8]. Furthermore, extended exposure to sunlight is associated with the development of nuclear and cortical cataracts, while posterior subcapsular cataract’s relation with sun exposure is not proven [9,10]. UVA and UVB’s, as well as blue light’s, association with cataract development was proven in experimental animal studies [11,12]. The connection between sun exposure and the formation of cataract is further enhanced by the evidence that the wearing of eye protection (sunglasses, hats and umbrellas) under sun exposure is associated with a lower risk of cataract [13].

According to our knowledge, we found only one study that investigated the association of age-related cataract with CM [14]. Sharma et al. proved a positive association between age-related cataract and skin cancer together with its subtypes, including premalignant lesions, in an older Australian population. However, no case–control studies have yet been published where cataract and its subtypes are compared in CM and healthy subjects. In Northern Europe, this is the first study that presents the distribution of different types of cataracts and their relationships with severe sunburns.

## 2. Materials and Methods

### 2.1. Study Design

A one-center hospital-based case–control study was performed after the approval of the Biomedical Research Ethics Committee (2021-06-09 No. BE-2-66). The case group consisted of 180 patients who were originally diagnosed with CM; diagnosis was established through histopathological evaluation. The control group consisted of 182 individuals, selected from subjects with no history of dermatological disorders. The inclusion criteria for the case group comprised subjects’ diagnoses of CM stages I–IV according to the 8th edition of the American Joint Committee on Cancer Melanoma staging system [15]. Inclusion criteria for the control group comprised dermatologically healthy subjects who had no skin disorders. In both the case and control groups, only adults who agreed to participate in the study were included [5].

### 2.2. Ophthalmological Examination

**Figure 1:**
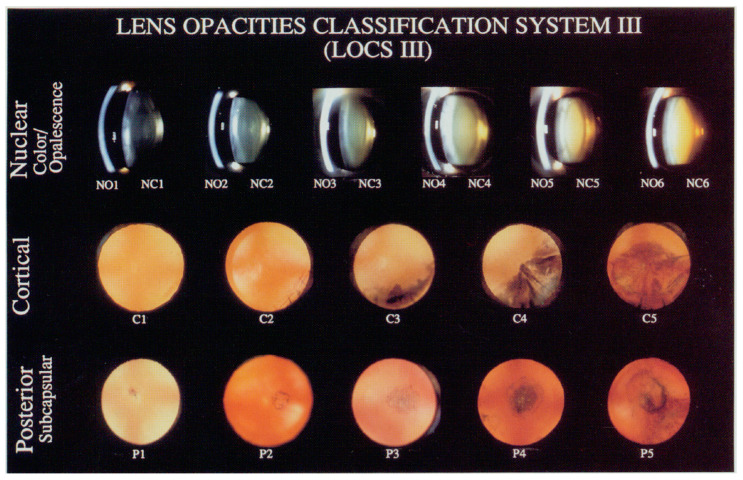
LOCS III scale is used to evaluate and grade lens opacifications and cataract. LOCS III is a set of standards prepared as slide for lens nuclear opalescence (NO), nuclear color (NC) and cortical (C) and posterior subcapsular (P) cataract evaluation. For each type, a grade from 1 to 5 or 6 is chosen, where a higher grade represents more advanced cataract; for example, NO1 to NO6, NC1 to NC6, C1 to C5 and P1 to P5 [16].

The examination of subjects included an evaluation of best-corrected visual acuity, a measure of intraocular pressure and a detailed examination using a slit lamp. Additionally, lens status and cataract formation were evaluated according to the Lens Opacities Classification System III (LOCS III) [16]. One experienced ophthalmologist (L.S.) evaluated lens status during examinations. One of six grades according to the slit lamp images was selected for nuclear opalescence (NO) and nuclear color (NC). Also, one of five grades was chosen for cortical (C) and posterior subcapsular (P) cataract evaluation according to retro illumination images, as illustrated in Figure 1 [16]. As we observed only sporadic cases with grades higher than 4, in the Results section, higher grades are shown combined. Both the left and right eyes were evaluated on the LOCS III scale, but the higher grade was chosen for the analysis if they did not match. Aphakic and pseudophakic eyes were not included in the analysis, as well as eyes with traumatic cataract or eyes with severe blunt trauma history.

### 2.3. Dermatological Examination

Skin examinations were carried out by 3 medical doctors trained by an experienced dermatologist (S.V.). Hair and eye color were recorded for each subject. Skin color assessment was performed on the left buttock using a 12-skin-tone panel color scale. Skin phototype (I–IV) was categorized according to Fitzpatrick based on questionnaire data of subjects’ responses to ultraviolet light [17]. For the counting of common melanocytic nevi (CMNs) in 26 body regions except for the buttocks and genitalia, we used the standardized protocol from our previous studies [5,14]. Freckles, solar lentigines and café au lait spots were excluded from the total number of CMNs. We additionally examined the outer surfaces of the body regions, in which a higher CMN density due to intermittently sun-exposed skin areas was found [18]. We analyzed the number of CMNs on the face, outer surfaces of the upper arms, lower arms and hands. The CMNs were classified into the following groups: 2 mm or smaller; from 2 mm to 5 mm; larger than 5 mm. Atypical melanocytic nevi (AMNs) were observed according to the ABCDE rule: asymmetry, border irregularity, non-homogeneous pigmentation, diameter >6 mm and erythema or elevation. Diagnosis of AMNs was established upon the fulfillment of at least 3 out of 5 clinical criteria.

### 2.4. Questionnaire Data

All participants in the study completed multiple questionnaires designed to gather comprehensive information regarding their sun-related behavior. Specifically, data were collected about behaviors such as whether they regularly wear sunglasses or apply sunscreen. In addition, participants were also asked to provide details about instances of sunburn they experienced during the last year, including their frequency and severity.

### 2.5. Data Analysis

Microsoft Office Excel was used for data entry. The data were analyzed using the 29.0 version of IBM SPSS Statistics software. Quantitative variables were detailed through the use of statistical measures such as mean and standard deviation (SD) or median, along with the range from the minimum to the maximum values. Qualitative variables were described by the frequency and relative frequency within the comparative samples. 

Before conducting parameter tests, verification of the variance distribution was undertaken. A comparison of the quantitative variables between the two groups was conducted using a Student’s *t*-test or Mann–Whitney test, while qualitative variables between the groups were assessed using Chi-square or Fisher’s exact tests. A multivariable binary logistic regression model was applied to assess the relationships between several independent variables and a dependent binary outcome. An age- and sex-adjusted odds ratio (AOR) was used to determine the association between the variables with a statistically significant level at a 95% confidence interval (CI). Statistical significance was determined at a threshold less than 5% (*p* < 0.05).

## 3. Results

The subjects in the case and control group were equally distributed by gender, age and age groups (Table 1). Skin type, hair color and familiar anamnesis of CM did not differ between groups. The average age of the subjects was 59.56 years in the case group and 57.58 years in the control group. In both the case and control group, a larger proportion of the subjects was female—62.8% and 62.1%, respectively. However, this larger proportion of females corresponded to the gender proportions of patients with CM registered in the Lithuanian health statistics databases [19]. Detailed group characteristics and comparisons between the groups are shown in Table 1.

In Table 2, the detailed CMN distribution on the outer skin surfaces of the CM subjects and controls is shown. We found that in the case group, CMNs of both 2–5 mm and >5 mm in diameter were more common on the face and on the outer surface of the upper arm, and 2–5 mm CMNs on the outer surface of the lower arm. However, we did not find significant differences between the groups in the assessment of CMNs on the outer surfaces of the hands.

When assessing the differences in lens opacification between the groups, we found that NO2 was more often observed in the control group at 46.1% than in the case group at 35.1%. In the case group, NO3 was confirmed for 46.0% of subjects and in the control group for 34.3%. When assessing the color of the lens, we found that NC2 was more common in the control group than in the case group, 45.5% and 31.6%, respectively. However, NC3 was more often found in the case group at 44.8% than in the control group at 32.6%. In the control group, C1 was detected in 47.8% and in the case group in 14.9% of subjects. However, the more-opacified cortical layers of the lens C2 were more often found in the case group, at 65.5%, than in the 34.3% of subjects in the control group. Posterior subcapsular layers were evaluated as P1 more often in the control group, 64.0%, than 21.8% in the case group. P2 was more often found in the case group than in the control group, at 69.5% and 27.0%, respectively. More detailed estimates of the LOCS III scale are given in Table 3.

A higher risk of CM was calculated for subjects with a higher number of total CMNs on their face (AOR = 1.25; 95% CI: 1.13; 1.39), outer surfaces of upper (AOR = 1.05; 95% CI: 1.02; 1.07) and lower (AOR = 1.04; 95% CI: 1.00; 1.09) arms and hands (AOR = 1.29; 95% CI: 1.02; 1.61). However, while evaluating CMNs bigger than 5 mm, increased CM was only observed on CMNs located in outer surface of upper arm.

Subjects with nuclear opacity (NO) grade ≥ 3 (AOR = 1.82; 95% CI: 1.15; 2.88), nuclear color (NC) grade ≥ 3 (AOR = 2.02; 95% CI: 1.29; 3.16) and cortex (C) grade ≥ 2 (AOR = 5.24; 95% CI: 3.11; 8.84), as well as a posterior subcapsular P ≥ 2 (AOR = 6.34; 95% CI: 3.93; 10.25), had a higher risk of CM than the controls.

The detailed results of multivariable binary logistic regression are shown in Table 4.

## 4. Discussion

No other perspective or case–control studies have analyzed the association of CM and cataract, despite the overlapping impact of sun exposure on both diseases. Our study shows that the lens nuclear opacity (NO), nuclear color (NC), cortical (C) layer and posterior subcapsular (P) layer grades according to LOCS III scale were higher in subjects with CM than in healthy controls. Patients with an LOCS III grade of NO ≥ 3 had a 1.82 times higher risk of CM, and patients with a NC grade ≥ 3 had a 2.02 times higher risk of CM. An LOCS III evaluation of C and P showed an increased risk of CM by 5.24 and 6.34 times, respectively.

A cross-sectional study in the Australian population reported that age-related cataract is positively associated with skin cancer and its subtypes, including premalignant lesions [14]. Another cross-sectional study in Israel reported that various skin cancers had an increased likelihood of prevalent cataract [8].

Several studies have reported an increased risk of cataract in subjects with prolonged sun exposure. One of these studies compared cataract-related blindness with socioeconomic status and estimated ground-level ultraviolet radiation exposure across all countries and revealed that long-term high-UV exposure mainly from sunlight amplifies the association of poor socioeconomic status with the burden of cataract-related blindness [20]. Another study analyzed the association between sun exposure and cataract by evaluating lens opacities with the LOCS III scale. This study showed a positive association between increasing sun exposure and nuclear and cortical cataract formation [9]. Tang et al. found that outdoor activity was an independent risk factor for cortical cataract but was not a risk factor for nuclear and posterior subcapsular cataracts. Their study results showed increased cortical cataract risk by 4.3% when outdoor activity time increased every one hour [21]. Also, one study in the Australian population revealed a strong positive association of occupational sun exposure with nuclear cataract [22]. A study in Taiwan revealed that wearing eye protection under sun exposure is associated with a lower risk of cataract [13].

We did not find similar articles of prospective studies in the scientific literature that analyzed the risk of CMNs and cataract formation. However, several studies have investigated the relationship between CMN count and CM risk. In our study, the number of CMNs of all sizes was higher in CM subjects than in controls. On the outer surface of the lower arms, only 2–5 mm diameter CMNs and the total number of CMNs was higher in subjects with CM than the controls. The number of CMNs on the face, the outer surface of the upper and lower arms, and the outer surface of the hands increased CM risk. Similar results are presented by other studies [23,24,25]. Although no studies have been conducted in Baltic countries and Eastern Europe to analyze risk factors for CM, a recent study in Sweden obtained similar results. Ivert et al. found that the risk of CM was higher in people who were exposed to more UV radiation (used sun beds, had a diagnosis of actinic keratosis or CMNs and were born in the South) [26].

UV radiation is a risk factor for both CM and cataract [11,12,27]. Therefore, when studying the relationship between CM and cataract, it would be challenging to design a study in which one group was exposed to less UV radiation. Thus, UV radiation should be considered a risk for both CM and cataract, but the common etiological factor makes it difficult to determine the relationship between cataract and CM.

In our study, we asked subjects about their sun-related behavior in the last year. Asking only about the previous years’ experience was chosen due to the different ages of the subjects and the fact that the subjects themselves would be subjective when evaluating their older experiences. Naturally, this is one of the limitations of this study. However, to correct this limitation, it would be appropriate to ask subjects about their sun-related behavior and conduct a long-term prospective study in which the time spent in the open air or the sun would be recorded. Although Lithuania has significantly fewer sunny days than other parts of the world, a high incidence of skin melanoma is observed [28]. It is unknown whether this may be due to society’s attitude to being less protective of sun exposure or to a longer time spent outdoors.

When analyzing the data of the questionnaire, our findings revealed that in the case group, the subjects were wearing sunglasses less often than in the control group. On the other hand, it showed that individuals who do not wear sunglasses are more prone to CM and cataract. Studies conducted by other authors confirm this [13]. Also, subjects in the case group were more likely to experience severe sunburns and suffer redness after sunburn, as well as more often having peeled skin or blisters after sunburn than the controls.

There were many studies about the association between sun exposure and CM or sun exposure and cataract, but only a few of them focused on the link between CM and cataract. So further research, especially different study designs, in this field would be beneficial to understand the relation between CM and cataract.

## Figures and Tables

**Table 1:** Characteristics of the subjects with CM (case group) and the controls.

Characteristics	Control Group n = 182	Case Group n = 180	*p*-Value
Sex, % (*n* ^1^)			0.892
Male	37.9 (69)	37.2 (67)	
Female	62.1 (113)	62.8 (113)	
Total	100.0 (182)	100.0 (180)	
Age (years), mean (SD ^2^)			
Male	57.97 (8.2)	60.70 (13.5)	0.155
Female	57.34 (7.83)	58.88 (11.89)	0.249
Total	57.58 (7.95)	59.56 (12.51)	0.072
Age groups (years), % (*n*)			0.974
≤50	23.6 (43)	22.8 (41)	
51–60	25.8 (47)	26.7 (48)	
≥61	50.5 (92)	50.6 (91)	
Skin color, % (*n*)			**<0.001**
Fair	29.5 (52)	48.6 (87)	
Medium	65.9 (116)	40.8 (73)	
Olive	4.5 (8)	10.6 (19)	
Skin type (Fitzpatrick scale), % (*n*)			0.061
Type I	15.3 (27)	21.7 (39)	
Type II	39.5 (70)	36.1 (65)	
Type III	30.5 (53)	21.1 (38)	
Type IV	14.7 (26)	21.1 (38)	
Hair color, % (*n*)			0.134
Light brown	51.7 (91)	62.0 (111)	
Dark brown	30.5 (54)	25.1 (45)	
Black	14.7 (26)	12.8 (23)	
Familiar anamnesis of CM, % (*n*)			0.541
Yes	2.2 (4)	3.3 (6)	
No	97.8 (178)	96.7 (174)	
Wears sunglasses, % (*n*)			**<0.001**
Yes	63.5 (115)	43.6 (78)	
No	36.5 (66)	56.4 (101)	
Severe sunburn, % (*n*)			
Did not have severe sunburn	69.8 (127)	10.0 (18)	**<0.001**
Had sunburn redness	14.8 (27)	23.3 (42)	**0.004**
Had peeled skin after sunburn	13.2 (24)	38.9 (70)	**<0.001**
Had skin blisters after sunburn	2.2 (4)	27.8 (50)	**<0.001**

^1^ *n*—the total number of individuals or observations in the sample; ^2^ SD—standard deviation; CM—cutaneous melanoma.

**Table 2:** The counted number of CMN distribution on outer skin surfaces between groups.

Area	Control Group n = 182	Case Group n = 180	*p*-Value
Face, mean (SD)			
Diameter 2–5 mm	0.23 (0.607)	1.12 (1.814)	**<0.001**
Diameter ≥ 5 mm	0.17 (0.733)	0.36 (0.837)	**0.024**
Total	0.4 (0.951)	1.48 (2.213)	**<0.001**
Outer surface of upper arm, mean (SD)			
Diameter 2–5 mm	1.39 (3.659)	3.52 (4.718)	**<0.001**
Diameter ≥ 5 mm	0.06 (0.354)	0.37 (1.246)	**0.002**
Total	1.45 (3.821)	3.89 (4.967)	**<0.001**
Outer surface of lower arm, mean (SD)			
Diameter 2–5 mm	0.34 (1.045)	1.57 (2.701)	**<0.001**
Diameter ≥ 5 mm	0.06 (0.42)	0.03 (0.18)	0.509
Total	0.4 (1.278)	1.61 (2.757)	**<0.001**
Outer surface of hands, mean (SD)			
Diameter 2–5 mm	0.07 (0.411)	0.13 (0.485)	0.246
Diameter ≥ 5 mm	0.04 (0.402)	0.01 (0.075)	0.273
Total	0.11 (0.626)	0.13 (0.489)	0.358

**Table 3:** Lens opacity evaluation by LOCS III scale grade distribution between groups.

LOCS III Grade	Control Group n = 182	Case Group n = 180	*p*-Value
Nuclear opacity (NO), % (*n* ^1^)			
1	11.2 (20)	6.3 (11)	0.104
2	46.1 (82)	35.1 (61)	**0.035**
3	34.3 (61)	46.0 (80)	**0.025**
≥4	8.4 (15)	12.6 (22)	0.197
Nuclear color (NC), % (*n*)			
1	11.2 (20)	6.9 (12)	0.157
2	45.5 (81)	31.6 (55)	**0.007**
3	32.6 (58)	44.8 (78)	**0.018**
≥4	10.7 (19)	16.7 (29)	0.101
Cortical (C), % (*n*)			
1	47.8 (85)	14.9 (26)	**<0.001**
2	34.3 (61)	65.5 (114)	**<0.001**
3	14.6 (26)	16.1 (28)	0.699
≥4	3.4 (6)	3.4 (6)	0.968
Posterior subcapsular (P), % (*n*)			
1	64.0 (114)	21.8 (38)	**<0.001**
2	27.0 (48)	69.5 (121)	**<0.001**
3	7.9 (14)	5.2 (9)	0.307
≥4	1.1 (2)	3.4 (6)	0.143

^1^ *n*—the total number of individuals or observations in the sample.

**Table 4:** Multivariable binary logistic regression predicting the risk factors of cutaneous melanoma.

	Odds Ratio	95% Confidence Interval		*p*-Value
		Lower Bound	Upper Bound	
Sex, female (reference)	0.97	0.64	1.49	0.892
Age	1.02	0.99	1.04	0.073
Age groups, ≤50 (reference)				
51–60	1.07	0.60	1.93	0.819
≥61	1.04	0.62	1.74	0.889
CMN numbers on face, mean *				
Diameter 2–5 mm	2.20	1.65	2.94	** <0.001 **
Diameter ≥ 5 mm	1.37	0.98	1.90	0.062
Total	1.25	1.13	1.39	** <0.001 **
CMN numbers on the outer surface of upper arm, mean *				
Diameter 2–5 mm	1.16	1.08	1.24	** <0.001 **
Diameter ≥ 5 mm	2.32	1.29	4.19	** 0.005 **
Total	1.05	1.02	1.07	** <0.001 **
CMN numbers on the outer surface of lower arm, mean *				
Diameter 2–5 mm	1.58	1.31	1.89	** <0.001 **
Diameter ≥ 5 mm	0.81	0.40	1.64	0.562
Total	1.04	1.00	1.09	** 0.047 **
CMN numbers on the outer surface of hands, mean *				
Diameter 2–5 mm	1.33	0.81	2.17	0.264
Diameter ≥ 5 mm	0.49	0.11	2.19	0.352
Total	1.29	1.02	1.61	** 0.030 **
Lens nuclear opacity (NO) evaluated by LOCS III scale, grade ≤ 2 (reference) *				
≥3	1.82	1.15	2.88	** 0.010 **
Lens nuclear color (NC) evaluated by LOCS III scale, grade ≤ 2 (reference) *				
≥3	2.02	1.29	3.16	** 0.002 **
Lens cortical (C) layers evaluated by LOCS III scale, grade 1 (reference) *				
≥2	5.24	3.11	8.84	** <0.001 **
Lens posterior subcapsular (P) layers evaluated by LOCS III scale, grade 1 (reference) *				
≥2	6.34	3.93	10.25	** <0.001 **
Wears sunglasses *	2.18	1.42	3.35	**<0.001**
History of severe sunburn; did not have severe sunburn (reference) *				
Had sunburn redness	10.91	5.44	21.85	** <0.001 **
Had peeled skin after sunburn	22.32	11.18	44.56	** <0.001 **
Had skin blisters after sunburn	86.21	27.67	268.59	** <0.001 **

* odds ratio adjusted by age and sex.

## Data Availability

The datasets used and/or analyzed during the current study are available from the corresponding author on reasonable request.
